# Impact of antibiotic therapy during a bedside percutaneous tracheotomy procedure in an ICU

**DOI:** 10.1186/cc14296

**Published:** 2015-03-16

**Authors:** E Brotfain, A Borer, L Koyfman, A Frenkel, S Gruenbaum, A Smolikov, A Zlotnik, M Klein

**Affiliations:** 1 Ben Gurion University of The Negev, Soroka Medical Center, Beer Sheva, Israel; 2 Yale University School of Medicine, New Haven, CT, USA

## Introduction

Percutaneous bedside tracheostomy (PBT) is a frequently done procedure in the ICU. PBT is a clean-contaminated procedure, and the duration of the procedure is 15 to 20 minutes depending on the physician's procedural skills. The rate of infectious complications and efficacy of perioperative therapy in reducing infections after PBT is currently unknown. Currently there have been no definitive recommendations for prophylactic antibiotic therapy before PBT in the ICU.

## Methods

All clinical and microbiological data were retrospectively collected and analyzed during the ICU stay before PBT performance and 72 hours after the PBT procedure from 110 patients in our ICU. Controls were defined as patients in whom the PBT procedure was performed in the ICU, with antibiotics administered 72 hours prior to and during the procedure (Group 1, *n *= 82). Cases were defined as patients in whom the PBT procedure was performed in the ICU without antibiotics administered 72 hours prior to and during the procedure (Group 2, *n *= 28). Secondary bacteremia, line sepsis and VAP during the 72 hours after PBT were considered infectious complications. Two-tailed *P *< 0.05 was considered to be significant.

## Results

No differences were found in age, gender, admission diagnoses, length of ICU stay and in-hospital mortality rate between the two study groups. Overall Gram-negative, Gram-positive and fungal flora were similar in both groups before and after PBT. Patients who received antibiotic therapy had a lower incidence of new ventilator-associated pneumonia (VAP) episodes (15/82 (18.2%) in Group 1 vs. 14/28 (50%) in Group 2, *P *< 0.001 (0.23, 0.87 to 0.13)) (Table 1). There were no differences in the incidence of bacteremia or line sepsis (Table 1).

**Table 1:** Clinical data of new infections 72 hours after the PBT procedure.

Infection	Group 1	Group 2	*P *value OR
Bacteremia	21/82(25.6%)	11/28(39.3%)	0.149
Colonization	67/82(82.7%)	23/28(82.1%)	0.718
Line sepsis	10/82 (12%)	4/28 (14.3%)	0.39
New VAP (%)	15/82(18.2%)	14/28 (50%)	0.001

## Conclusion

Our findings highlight the importance of conducting a prospective randomized control trial to better understand the role of antibiotic prophylaxis in PBT.

